# Hesperidin and Naringin Improve Broiler Meat Fatty Acid Profile and Modulate the Expression of Genes Involved in Fatty Acid β-oxidation and Antioxidant Defense in a Dose Dependent Manner

**DOI:** 10.3390/foods10040739

**Published:** 2021-03-31

**Authors:** Ariadne L. Hager-Theodorides, Theofilos Massouras, Panagiotis E. Simitzis, Katerina Moschou, Evangelos Zoidis, Eleni Sfakianaki, Katerina Politi, Maria Charismiadou, Michael Goliomytis, Stelios Deligeorgis

**Affiliations:** 1 Laboratory of Animal Breeding and Husbandry, Department of Animal Science, Agricultural University of Athens, 75 Iera Odos, 11855 Athens, Greece; pansimitzis@aua.gr (P.E.S.); lenasfakianakis@gmail.com (E.S.); katerinapoliti@aua.gr (K.P.); charisma@aua.gr (M.C.); mgolio@aua.gr (M.G.); sdel@aua.gr (S.D.); 2 Laboratory of Dairy Science and Technology, Department of Food Science and Human Nutrition, Agricultural University of Athens, 75 Iera Odos, 11855 Athens, Greece; theomas@aua.gr (T.M.); kmoschou@yahoo.gr (K.M.); 3 Laboratory of Nutritional Physiology and Feeding, Department of Animal Science, Agricultural University of Athens, 75 Iera Odos, 11855 Athens, Greece; ezoidis@aua.gr

**Keywords:** citrus flavanones, antioxidants, lipid metabolism, fatty acids, hepatic gene expression, FA beta-oxidation, glutathione

## Abstract

The beneficial properties of the flavanones hesperidin and naringin as feed additives in poultry have lately been under investigation. In broilers, both flavanones have been shown to exhibit antioxidant properties while their individual effects on fatty acid (FA) composition and the underlying molecular mechanisms of their activity have not been explored. Here, we studied their effects on broiler meats’ FA profiles and on the expression of genes related to lipid metabolism, antioxidant defense and anti-inflammatory function. The experimental design comprised six treatment groups of broilers, each supplemented from day 11 until slaughter at 42 days with hesperidin, naringin or vitamin E, as follows: the E1 group received 0.75 g of hesperidin per kg of feed, E2 received 1.5 g hesperidin/kg feed, N1 received 0.75 g naringin/kg feed, N2 received 1.5 g naringin/kg feed, vitamin E (VE) received 0.2 g a-tocopheryl acetate/kg feed, and the control group was not provided with a supplemented feed. The VE treatment group served as a positive control for antioxidant activity. An analysis of the FA profiles of the abdominal adipose tissue (fat pad), *major pectoralis* (breast) and *biceps femoris* (thigh) muscles showed that both hesperidin and naringin had significant effects on saturated FA (SFA), polyunsaturated FA (PUFA) and omega n-6 content. Both compounds reduced SFA and increased PUFA and n-6 content, as well as reducing the atherogenicity and thrombogenicity indices in the breast muscle and fat pad. The effects on the thigh muscle were limited. An analysis of gene expression in the liver revealed that naringin significantly increased peroxisome proliferator-activated receptor alpha (*PPARα*), Acyl-CoA oxidase 1 (*ACOX1*) and glutathione disulfide reductase (*GSR*) expression. In the breast muscle, both hesperidin and naringin increased fatty acid synthase (*FASN*) expression and hesperidin increased the expression of adiponectin. In brief, both hesperidin and naringin supplementation beneficially affected FA profiles in the breast meat and fat pad of broiler chicken. These effects could be attributed to an increase in FA β-oxidation since the increased expression of related genes (*PPARα* and *ACOX1*) was observed in the liver. Furthermore, the antioxidant activity of hesperidin and naringin previously observed in the meat of broilers could be attributed, at least partly, to the regulation of antioxidant defense genes, as evidenced by the increased *GSR* expression in response to naringin supplementation.

## 1. Introduction

The poultry industry worldwide is in search of bioactive and cost-effective compounds that can improve product quality and human health-promoting attributes. The potential benefits for poultry production that can be derived from dietary supplementation with plant flavonoids have recently been under investigation with so far encouraging results, especially for fat quality and antioxidant function [1]. Amongst flavonoids, hesperidin and naringin (flavanones that are abundant in citrus fruits) are potent antioxidants, possess anti-inflammatory properties, improve metabolic syndrome disease symptoms and modulate lipid metabolism [2]. 

Some of the desirable properties of broiler meats as regards fat quality are reduced fat content and favorable fatty acid composition, e.g., increased poly-unsaturated/saturated fatty acid (PUFA/SFA) ratio and reduced omega n-6/n-3 ratio and atherogenicity (AI) and thrombogenicity (TI) indices. Hesperidin has been shown to decrease muscle fat content in broilers [3], increase PUFA, improve n-6/n-3 and PUFA/SFA ratios in breast meat, and decrease serum and muscle cholesterol and triglyceride levels [4]. Furthermore, hesperidin and naringin were found to reduce cholesterol content in layer hens’ egg yolk [5,6,7] and to reduce serum cholesterol levels in layer hens [6,7]. In humans, flavonoids including hesperidin have been shown to improve metabolic syndrome health indices [8]. 

Another important quality parameter for poultry meat is oxidative stability [9]. Dietary supplementation with hesperidin has been shown to increase broiler meat antioxidant capacity during storage [10,11,12,13] and to improve antioxidant defense function in the plasma [4] and liver [3]. Naringin has also been found to reduce the oxidative deterioration of stored broiler meat [11]. Naringin and/or hesperidin, or their aglycones naringenin and hesperetin, have been found to alleviate the symptoms of induced oxidative stress in rodents and rabbits, in the context of human disease animal model systems such as metabolic disorder, diabetes and liver injury, and in human cell lines [14,15,16,17,18,19,20,21]. 

Citrus flavanones are also known to possess anti-inflammatory properties [2,22]. In chicken, hesperidin has been found to exert immunomodulatory functions, increasing antibody titers following immunizations, improving heterophil adhesion, elevating responses to cutaneous basophilic hypersensitivity tests and increasing phagocytic activity following lipopolysaccharide challenge [23,24]. 

Hesperidin, naringin and their aglycones seem to mediate the effects described above on lipid metabolism, antioxidant defense and immune regulation via the modulation of genes involved in relevant pathways [25,26,27]. Their effects on gene expression have been studied in many tissues and cell types, in vivo and in vitro, and under different physiological conditions, mainly in rodents, rabbits and human cell lines. The beneficial effects of the two flavonoids on metabolic disease have been linked to an increased hepatic expression of genes involved in fatty acid (FA) β-oxidation (such as *PPARα*, *PPARγ*, *ACOX*, *CPT1A*) and the decreased expression of genes involved in lipogenesis and lipid metabolism (e.g., *FAS*, *Srebf1*, *ACC*). Their antioxidant activity seems to be exerted via the downregulation of pro-apoptotic (e.g., *Casp3*, *Casp9*, *BAX*) and the upregulation of the anti-apoptotic (e.g., *BCL-2*) and antioxidant defense system (e.g., *SOD*, *CAT*, *GSH-P*, *GPx*, *GR*) genes. Their anti-inflammatory properties are linked to the modulation of genes involved in pro- and anti-inflammatory processes, such as *iNOS*, *COX-2*, *Nrf2*, *NFκB*, *TGFβ* and *IL10*. A comprehensive list of genes per functional category and corresponding references are presented in the supplementary material (Supplementary Table S1).

In this study we investigated the effect of hesperidin and naringin supplementation on broiler meat’s FA profile. Furthermore, we provide novel data on the expression of genes related to the known effects of the two flavanones on antioxidant defense, lipid metabolism, and inflammation.

## 2. Materials and Methods

### 2.1. Animals and Experimental Design 

In this study, 240-day-old Ross 308 broiler chickens, obtained from a commercial hatchery, as hatched, were housed in a controlled environment. Animal management and feed are described in [11]. The 240 broiler chickens were equally allocated to 6 dietary treatment groups and 2 pens per treatment group. The six treatment groups were: N1 and N2, supplemented with 0.75 and 1.5 g naringin (Alfa Aesar GmbH & Co KG, Kandel, Germany) per kg of feed, respectively; E1 and E2, supplemented with 0.75 and 1.5 g hesperidin (TSI Europe NV, Zwijndrecht, Belgium) per kg of feed, respectively; control (C) with no feed additive; and vitamin E (VE) supplemented with 0.2 g a-tocopheryl acetate (vitamin E) (DSM Nutritional Products Hellas, Athens, Greece) per kg of feed. The VE treatment group served as a positive control for antioxidant activity and the level of supplementation was determined according to previously published data [28]. Feed additives were supplemented from the 11th day of age until slaughter at 42 days. 

### 2.2. Fatty Acid Profile Analysis

#### 2.2.1. Lipid Extraction

Total lipids were extracted from abdominal adipose tissue (fat pad), the breast (*pectoralis major*) and the thigh (*biceps femoris*) muscle from 10 birds per treatment group according to Folch et al. [29]. Afterwards, the organic phase was dried under reduced pressure with a rotary evaporator. The lipid extract was weighted, the percentage fat content was determined, and it was then subjected to transmethylation.

#### 2.2.2. Transesterification and Gas Chromatographic Analysis

Direct transesterification on lipid extract was performed following [30] with minor modification. Briefly, a quantity between 100 and 150 mg of lipid extract was directly methylated with 2 mL of 0.5 M sodium methylate at 50 °C for 30 min, followed by 2 mL of 140 g L^−1^ boron trifluoride in methanol (BF3) at 50 °C for 30 min. Then, 2 mL of hexane was added and the upper hexane phase containing the fatty acid methyl esters (FAMEs) was transferred to gas–liquid chromatography (GLC) auto-sampler vials and analyzed in duplicate. 

FAMEs were separated by GLC using a Shimadzu gas chromatograph (model GC-17A, Columbia, MD, USA) with a Shimadzu GC-2014 GC AOC-20i auto injector, equipped with a flame ionization detector (FID). The FA composition of the FAME was determined by capillary GC on a SP-2560 capillary column (75 m × 0.18 mm I.D., 0.14 μm; Supelco Inc., Bellefonte, PA, USA). The flow rate of carrier gas (Helium) was 1 mL∙min^−1^, the injection temperature was 250 °C and the detector temperature was 270 °C. The injection volume was 1 μL (split 1:50). The temperature program was as follows: The initial temperature was held at 75 °C for 5 min after injection and then programmed to increase at 5 °C/min to 150 °C, to hold for 5 min, then to increase to 220 °C at 7 °C/min and hold for 20 min. Fatty acid peaks were recorded and integrated using a Shimadzu GC solution software (Shimadzu Corporation, Kyoto, Japan). Individual fatty acids were identified by comparing their retention times with known fatty acid methyl ester standards (Supelco 37 Component FAME Mix, purchased from Sigma-Aldrich, Taufkirchen, Germany). The individual FA content was expressed as weight percentage (g∙100 g^−1^ of total FA). SFA, PUFA, MUFA, n-6 and n-3 were calculated as the sum of the percent content of all saturated, polyunsaturated, monounsaturated, n-6 and n-3 FA, respectively. PUFA/SFA and n-6/n-3 ratios were calculated by dividing PUFA by SFA and n-6 by n-3, respectively. The atherogenicity and thrombogenicity indices were calculated with the following formulas [31]: AI = (12:0 + 4 · 14:0 + 16:0)/(Sum MUFA + Sum PUFA), TI = (14:0 + 16:0 + 18:0)/[0.5 · Sum MUFA + 0.5 · Sum (n-6) PUFA + 3 · Sum (n-3) PUFA + (n-3/n-6)]. 

### 2.3. RNA Extraction and cDNA Synthesis 

Samples from the liver, breast (*pectoralis major*) muscle and abdominal adipose tissue (fat pad) from 4 (liver, fat pad) or 6 (muscle) animals per dietary group (C, E1, E2, N1, N2 and VE) were collected post-mortem at 42 d of age, snap-frozen in liquid nitrogen and stored at −80 °C until the extraction of RNA. RNA was extracted using the QIAzol^®^ lysis reagent (Qiagen, Hilden, Germany) and according to the manufacturer’s instructions. Briefly, approximately 20 mg of frozen liver tissue was homogenized in 500 μL QIAzol^®^ lysis reagent and spun at 12,000× *g* for 10 min at 4 °C. Supernatant was mixed with 0.1 mL chloroform and incubated at room temperature for 5 min. The mixture was spun at 12,000× *g* for 15 min at 4 °C and the upper aqueous phase was mixed with 0.25 mL isopropanol and incubated on ice for 2 min and at room temperature for 10 min, then was spun at 12,000× *g* for 10 min at 4 °C. The pellet was washed with 0.5 mL 70% ethanol and was then resuspended in 50 μL dH_2_O. The RNA preparations were then treated with DNAseI (TAKARA Bio INC, Shiga, Japan) according to the manufacturer’s recommendations to eliminate gDNA contamination. RNA concentration and purity were assessed by spectrophotometry on a Quawell Q5000 micro volume cuvette free spectrophotometer. The synthesis of first strand cDNA for the quantitative (q)PCR arrays was performed using 500 ng total RNA with the RT^2^ first strand kit (Qiagen, Hilden, Germany) and according to the manufacturer’s protocol. The synthesis of cDNA for single-gene qPCR was performed with the PrimeScript RT-PCR Reagent Kit (TAKARA Bio INC, Shiga, Japan) using 1 μg total RNA in 20 μL reactions according to the manufacturer’s instructions.

### 2.4. Quantitative (q)PCR and PCR Arrays 

Custom RT^2^ Profiler PCR arrays (Qiagen, Hilden, Germany) were designed to include 36 genes related to antioxidant activity and apoptosis, lipid metabolism, and anti-inflammatory responses, 5 housekeeping genes for normalization of the expression, 1 genomic DNA contamination-negative control, 3 reverse transcription, and 3 PCR-positive controls (Table 1). The genes included in the array were chosen based on the available published data for the effect of hesperidin, naringin and their aglycones on the expression of genes in the liver (Supplementary Table S1). Each well in a 96-well PCR plate contained the primers for the amplification of one of the 41 transcripts or controls. With each PCR plate, the gene expression in two liver samples was assayed. The RT^2^ SYBR Green ROX qPCR mastermix (Qiagen, Hilden, Germany) was used to prepare 25 μL quantitative reverse transcription (qRT)-PCR reactions that were performed, according to the manufacturer’s guidelines, in an ABI 7500 thermal cycler (Applied Biosystems, ThermoFisher Scientific, Waltham, MA, USA) under the following thermal program: initial denaturation/activation for 10 min at 95 °C, 40 cycles of 15 s at 95 °C and 1 min at 60 °C. The cycles were followed by a melt curve analysis. 

Threshold fluorescence was manually defined using the log view, above the background signal and within the lower half of the linear amplification phase of the amplification plot. Threshold cycle (C_T_) values were exported for all wells and quality control analysis was performed using the SABiosciences PCR array data analysis excel template. The expression of each gene (GOI) was normalized using the geometric mean of the expression of the housekeeping genes (HKG) ($\frac{2^{-C_{T\left(GOI\right)}}}{2^{-C_{T\left(HKG\right)}}}=2^{-∆C_{T}}$). Fold differences in expression levels for each gene in the treatment groups (expt), relative to the control group’s mean expression (ctrl), were determined using the formula: $\frac{2^{-C_{T\left(expt\right)}}}{2^{-C_{T\left(ctrl\right)}}}=2^{-∆∆C_{T}}$. $2^{-∆∆C_{T}}$ values for each sample were extracted and submitted to statistical analysis using the SAS software (see “Statistical analysis” section).

Quantitative (q)RT-PCR was also performed to assess the expression of fatty acid synthase (*FASN*), peroxisome proliferator-activated receptor gamma (*PPARγ*), and adiponectin (*ADIPOQ*) in the breast muscle and fat pad. The actin beta (*ACTB*) gene was used as the internal control (housekeeping) gene for normalization. The primers used were as follows: *ACTB*—chACTB_F CGAGGCCCTCTTCCAGCCATCTTT and chACTB_R CACCAGACAGCACTGTGTTGGC; *ADIPOQ*—chAdipoQ_F CCAGGTCTACAAGGTGTCA and chAdipoQ_R CCATGTGTCCTGGAAATCCT; *PPARγ*—chPPARg_F TGTTGATTTTTCAAGCATTTCTTCACCACA and chPPARg_R AGGGAGGAGAAGGAGGCTCCAT; *FASN*—chFASN_F GGCTTGAGTTGGCACAGTGGCTA and chFASN_R CTTGGATTCCCAGCGCCTTCCA. All primers were designed so as to avoid genomic amplification (either across exon–exon boundaries or each primer of a pair was designed at different exons). Reactions were prepared with the KAPA SYBR FAST qPCR Master Mix (2X) Universal (KAPA Biosystems, Boston, Massachusetts, United States), at a 10 μL final volume using 1 μL cDNA (ca 20 ng total RNA) and 200 nM final concentration of each primer. The qPCR thermal protocol used was as follows: 1 cycle of 95 °C for 3 min, 40 cycles of 95 °C for 5 s, 60 °C for 30 s, and a final cycle at 60 °C for 30 s. This was followed by a melt–curve analysis to assess the specificity of the amplification. 

The efficiencies of the reactions for all genes were between 90 and 110%, and the correlation coefficient between the threshold cycle and the log(Quantity) for the standard curve was >0.990. The raw data were analyzed with the ABI 7500 software and the mRNA abundance (quantity) was calculated relative to the standard curve obtained from serial dilutions, and was included in each qPCR run. The normalized expression levels of a target gene in each sample were estimated as the ratio of the test gene quantity divided by the respective quantity of the ACTB gene. The relative normalized expression in each sample was estimated as the ratio of the normalized expression divided by the mean normalized expression of the same gene in the control group. 

### 2.5. Statistical Analysis 

Statistical analysis was performed using SAS Studio (SAS University edition) and the mixed models task. The treatment effect on FA and the indices was assessed using each test parameter as the dependent variable and the treatment as the explanatory classification variable with fixed effect. Pairwise comparisons were performed between treatments and Tukey adjustment was used for multiple comparisons correction. 

To assess the effect of hesperidin (E), naringin (N) and vitamin E (VE) on gene expression, the relative/normalized gene expression from the qPCR arrays, and the single gene qPCRs, the gene expression was assigned as the dependent variable, and E, N and VE were assigned as the classification variables with fixed effects. E and N had three class levels (0, 1, 2) and VE had two class levels (0, 1). The linear dose effects of E and N on FA and gene expression were assayed by assigning E and N as the explanatory continuous variables. Mean differences and treatment effects were considered significant for *p* < 0.05 and showed a trend towards difference at 0.10 > *p* > 0.05. 

### 2.6. Ethics Statement

This study was carried out in strict accordance with the guidelines of “Council Directive 86/609/EEC regarding the protection of animals used for experimental and other scientific purposes”. The protocol was approved by the Research Ethics Committee of the Department of Animal Science and Aquaculture of the Agricultural University of Athens (approval document no 20/20032013). All efforts were made to minimize animal suffering.

## 3. Results

### 3.1. Effects of Hesperidin, Naringin and Vitamin E on the Fatty Acid Profiles of Breast and Leg Muscle and Fat Pad

The intramuscular fat contents and FA profiles of the *pectoralis major* (breast) and *biceps femoris* (thigh) muscles and the abdominal fat pad were assessed in 10 animals per experimental group (Table 2, Table 3, Table 4 and Table 5). No differences in the percentage of intramuscular fat in the breast or thigh were observed between treatment groups (Table 2, *p* > 0.05).

In the breast intramuscular fat (Table 3), hesperidin (E) and naringin (N) supplementation significantly reduced SFA (by 5–7%), and increased the PUFA (by 8–10%) content and the PUFA/SFA ratio compared to the control diet (*p* < 0.05). Hesperidin and naringin were supplemented at two different levels (0.75 and 1.5 g per kg or feed), based on previous experimental data from our group for hesperidin supplementation [10], to investigate possible dose-dependent effects. A significant linear dose–response to both E and N supplementation was observed in SFA, PUFA, and PUFA/SFA ratio (*p*-linear < 0.01). No effect was observed on monounsaturated FA (MUFA) (*p* > 0.05). In addition, total n-6 levels were significantly increased (by 7.5–12%), and AI and TI were favorably affected by both E and N in a dose-dependent manner (*p*-linear < 0.01). Total n-6 was significantly increased in all E and N treatment groups, while AI was reduced in the E1, N1 and N2 groups and TI was reduced in the E1, E2 and N2 groups. The observed reduction in total SFA levels can be accounted for mainly by the reduced caproic (C6:0, E1 only), palmitic (C16:0, E1 and N2) and stearic (C18:0, E and N1) acids contents. The total PUFA and n-6 increase can be attributed to the increased content of linoleic acid (18:2n-6) (*p* < 0.05 and *p*-linear < 0.01).

The effects of the two flavonoids in the thigh (Table 4) were limited to a significant reduction in docosahexaenoic acid (DHA, C22:6n-3) in all treatment groups and increases in n-6/n-3 ratio in treatment groups E1, N1 and N2 compared to the control (*p* < 0.05). The response to naringin supplementation followed a linear dose–response in both cases, while the response to hesperidin was linear in the case of DHA only (*p*-linear < 0.01). 

In the fat pad (Table 5), similar to the effect in the breast, E and N significantly increased the contents of total PUFA (by 8.5–11%) and n-6 fatty acids (by 9–10%), as well as the PUFA/SFA ratio, in a dose-dependent manner (*p*-linear < 0.01). The PUFA, PUFA/SFA and n-6 in all E- or N-supplemented groups differed significantly from the control (*p* < 0.05).

Hesperidin significantly affected oleic (C18:1), linoleic and α-linolenic (ALA, C18:3n-3) acids in a linear dose-dependent manner (*p*-linear < 0.05). The trend of a linear dose effect of naringin on oleic acid was observed (0.05 < *p*-linear < 0.10), and this FA was significantly reduced in N1 compared to the control (*p* < 0.05). Supplementation with naringin linearly increased linoleic acid and ALA content (*p*-linear < 0.05). Linoleic acid was increased in both the N1 and N2 groups, while ALA increased significantly in E2 only (*p* < 0.05). In addition, the total MUFA was significantly reduced by hesperidin supplementation compared to the control (E1 and E2 *p* < 0.05 and *p*-linear < 0.01). 

Vitamin E diet supplementation was used in this experiment as a positive control for antioxidant activity. In the breast meat, VE reduced SFA content (palmitic acid and total SFA), increased eicosapentaenoic acid (EPA, C20:5n-3), total n-3 and PUFA/SFA ratio, and improved AI and TI compared to the control diet (*p* < 0.05, Table 3). In the thigh meat, DHA was reduced and n-6/n-3 ratio increased in VE compared to control (Table 4). In the fat pad, oleic acid and total MUFA were reduced, while the linoleic acid, total n-6, PUFA and PUFA/SFA ratio were increased (*p* < 0.05, Table 5). 

A few differences were observed between the E and N treatment groups compared to VE in the thigh and fat pad FA profiles (Table 4 and Table 5). In the thigh, myristic acid and DHA were increased in N2 and E2, respectively, compared to VE, while in the fat pad arachidonic acid (C20:4n-6) was lower in the VE compared to the E1 group only. 

In the breast, while all three supplements (E, N and VE) reduced SFA content compared to control, VE mainly reduced palmitic acid, and E and N reduced both palmitic and stearic acids. The stearic acid content was thus found to be significantly reduced in the breast of E and N compared to VE-treated animals (*p* < 0.05). Furthermore, the EPA and total n-3 content were lower, while linoleic acid, total n-6 and the n-6/n-3 ratio were increased in E and N compared to VE (*p* < 0.05, Table 3). 

### 3.2. Effects of Hesperidin and Naringin on Gene Expression in the Liver 

**Figure 1:**
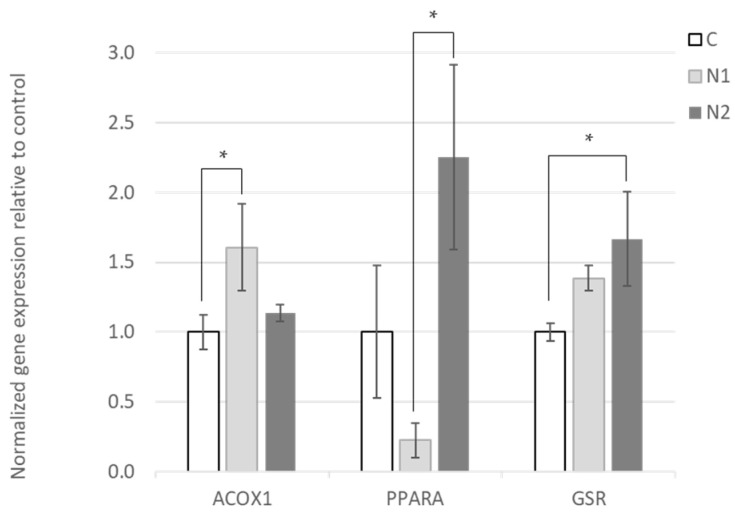
Effect of naringin on the hepatic expression of Acyl-CoA oxidase 1 (*ACOX1)*, peroxisome proliferator-activated receptor alpha (*PPARα*) and glutathione disulfide reductase (*GSR*) genes relative to control. Graph bars represent mean normalized gene expressions in the livers of animals that received C (no supplemented control), N1 (0.75 g naringin/kg feed) and N2 (1.5 g naringin/kg feed) diets relative to mean normalized expression in the control group. Expression was normalized with the geometric mean of four housekeeping genes. Sample size *n* = 4. Error bars represent SEM and * denotes statistically significant difference between means (*p* < 0.05). The effect of naringin on *GSR* expression is dose-dependent (*p*-linear = 0.008).

The hepatic expression of genes involved in biological processes related to oxidative regulation, apoptosis, fatty acid metabolism, lipid metabolism and inflammation was compared between treatment groups (Supplementary Table S2). A total number of 36 genes was selected based on extensive literature data mining to identify genes reported to be differentially expressed in the livers of animals supplemented with naringin, hesperidin, their aglycones naringenin and hespretin, or citrus fruit extracts (Supplementary Table S1). No significant effects of hesperidin or vitamin E on hepatic gene expression were observed (*p* > 0.05). On the contrary, naringin was found to significantly affect the expression of peroxisome proliferator-activated receptor alpha (*PPARα*, *p* < 0.05), Acyl-CoA oxidase 1 (*ACOX1*, *p* < 0.05) and glutathione disulfide reductase (*GSR*, *p* < 0.05) genes (Supplementary Table S2 and Figure 1). The trend of a positive linear dose–response relationship between naringin and *PPARα* expression was detected (*p*-linear = 0.068), as well as a significant increase in the expression in N2 compared to N1 (*p* < 0.05). The expression of *ACOX1* was significantly increased in N1 compared to the control (*p* < 0.05, Figure 1). A linear dose–response trend to naringin on the expression of *GSR* was observed (*p*-linear < 0.01), and the expression of the gene was significantly increased in N2 compared to the control (*p* < 0.05, Figure 1).

### 3.3. Expression of FASN, PPARγ and ADIPOQ Genes in the Fat Pad and Breast Muscle

The level of transcription of fatty acid synthase (*FASN*), peroxisome proliferator-activated receptor gamma (*PPARγ*) and adiponectin (*ADIPOQ*) genes was quantified in the breast muscle and fat pad (Table 6). No significant effects of hesperidin, naringin or vitamin E were detected on the expression of genes tested in the fat pad except an increasing trend for the expression of *FASN* in E1 group (*p* = 0.077). 

**Figure 2:**
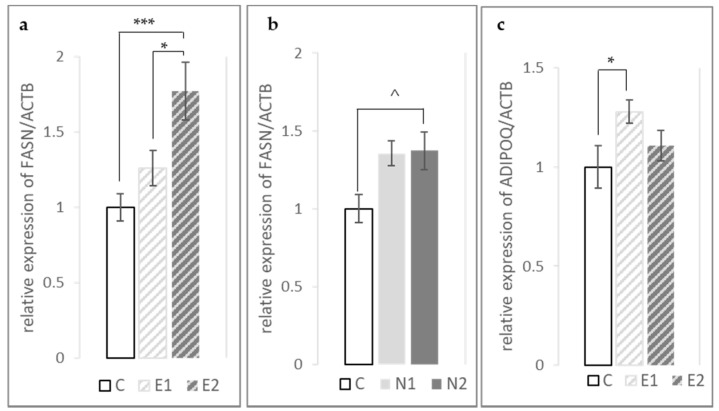
Relative expression of *FASN* and *ADIPOQ* genes in *pectoralis major* muscle. The expression of each gene shown is the mean of 6 biological replicates, and each sample is normalized for the corresponding ACTB expression and relative to the mean normalized expression in the control group. (**a**,**b**). Expression of *FASN* in C-E1-E2 and C-N1-N2 treatment groups, respectively. E1 (0.75 g of hesperidin per kg of feed), E2 (1.5 g hesperidin/kg feed), N1 (0.75 g naringin/kg feed), N2 (1.5 g naringin/kg feed) and C (control, no supplementation). Hesperidin’s effect on *FASN* expression: *p* = 0.0003, *p*-linear < 0.0001. Naringin’s effect on *FASN* expression: *p* = 0.06, *p*-linear = 0.01. (**c**). Expression of *ADIPOQ* in C-E1-E2 treatment groups. Hesperidin’s effect on *ADIPOQ* expression: *p* = 0.05. *p*-linear non-significant. Statistical differences between means are shown (* *p* < 0.05, *** *p* < 0.001, ^ *p* < 0.10).

On the contrary, a significant linear dose–response (to hesperidin and naringin) on the expression of *FASN* was observed in the breast. *FASN* expression increased with increasing hesperidin or naringin supplementation level (*p*-linear values < 0.0001 and 0.01, respectively, Figure 2a,b). Furthermore, a significant increase was observed in *ADIPOQ* expression in E1 compared to control (*p* < 0.05, Figure 2c).

## 4. Discussion

There is mounting evidence for the benefits of using plant flavonoids in poultry production, including their positive effects on meat fatty acid composition and oxidative stability [1,32]. Here, we found that the citrus flavanones hesperidin and naringin beneficially modulated the fatty acid profile of broiler chicken meat and fat pads (Table 3, Table 4 and Table 5). These effects were more pronounced in the breast meat and fat pad compared to thigh muscle. In the breast meat and fat pad, the PUFA and n-6 contents and PUFA/SFA ratio were increased. In addition, in the breast, the content of SFA was reduced. While the two compounds exhibited beneficial effects on meat FA composition, they had no adverse effects on growth performance [11]. The present study is the first to report the effects of the two flavanones on the FA profile of the fat pad and thigh muscle. The FA composition and transcriptional activity were examined in the fat pad, as in chicken it is an important site of FA storage and reflects the fat content of the animal [33,34]. Similar effects to those found here on the FA profile of breast meat have been reported following the supplementation of broilers with a mixture of hesperidin and genistein [4]. With increasing flavonoid mix concentrations, a reduction in SFA and MUFA was observed and an increase in PUFA in the breast meat was observed. Consistently with these results, the inclusion of orange (as yet unpublished data from our research group) or citrus [35] pulp in the broiler diet has been reported to beneficially affect FA profile. Both orange and citrus pulp increased PUFA content, while citrus pulp additionally reduced SFA and increased the PUFA/SFA ratio in broiler breast meat [35]. 

Metabolic disorders are currently a major human health issue worldwide. Apart from macronutrients, the quality of fat in the diet, including total SFA, PUFA and n-6 content, affects the development of metabolic disorder-related diseases. In addition to individual FA categories, PUFA/SFA, n-6/n-3 ratios and the atherogenicity and thrombogenicity indices have been proposed to predict the combined effects of different dietary factors on cardiovascular diseases [31]. Furthermore, total SFA and AI in the diet have been found to be significantly correlated with the atherogenic index of the plasma [36], an important index for atherosclerosis risk [37]. Here, we report a significant and dose-dependent beneficial effect of hesperidin, naringin and vitamin E supplementation on AI and TI in the breast muscle. Thus, in the present study we found that both hesperidin and naringin had significant and dose-dependent beneficial effects on various factors of fat quality related to human health, mainly in the breast muscle and fat pad. Consistently with previous reports, many of these effects were also observed following vitamin E supplementation [38,39], but they were more pronounced in the case of the two flavanones. 

In chicken, the liver is the main site of fatty acid biosynthesis [40,41]. To study the molecular mechanism underlying the observed effects on FA profile, we quantified the expressions of a number of genes involved in lipid metabolism in the liver (Table 1 and Table S2), breast muscle and fat pad (Table 6). We found that the hepatic expression of two genes involved in FA β-oxidation, *PPARα* and *ACOX1*, was significantly increased by naringin supplementation. PPARα is a ligand-activated transcription factor that regulates lipid metabolism, and in particular peroxisomal β-oxidation of FA. The activation of PPARα promotes the uptake, utilization, and catabolism of FA via the upregulation of genes involved in FA transport, binding and activation [42]. ACOX1, the first enzyme of the FA β-oxidation pathway, is a transcriptional target of PPARα. There are no reports on the effects of naringin on lipid metabolism-related genes in chicken. Nevertheless, consistently with our findings, quercetin, another plant flavonoid, was reported to increase the expression of PPARα in the livers of broilers [43]. 

The supplementation of the diet of metabolic disorder-affected mice with naringin and/or its aglycone naringenin has been shown to reduce the hepatic expression of genes related to de novo FA synthesis, and increase the expression of FA β-oxidation genes [44,45,46]. In these animal models, the effects of metabolic disease, such as hyperlipidemia and hypercholesterolemia, were attenuated by naringin/naringenin. Furthermore, similar effects were observed in the liver of naringin/naringenin-treated healthy mice [47], rats [48], and human and rat hepatic cell lines [49]. In all the above cases, the activation of FA β-oxidation was evident by the upregulation of *PPARα* and/or *ACOX1* gene expression, consistently with our findings, with the exception of the findings of Ke et al. [45] who, despite observing an upregulation of FA oxidation, detected reduced *ACOX1* expression. 

In the breast muscle, we found that *FASN* expression was increased significantly by both hesperidin and naringin in a dose-dependent manner (*p*-linear < 0.001 and 0.01, respectively). In contrast to our findings, the hepatic expression of *FASN* has been shown to be downregulated in response to hesperidin and naringin supplementation [44,46,49,50,51,52]. Nevertheless, it has been observed that effects on *FASN* expression may differ between the liver and skeletal muscle. A similar increase in *FASN* expression in the muscle in conjunction with reduced SFA and increased PUFA content was detected in broilers treated with *Aspergillus awamori* [53]. Interestingly, *FASN* mRNA expression was upregulated in the muscle tissue of naringenin-supplemented (3% *w*/*w*) mice relative to control, but did not reach significance (*p* = 0.07) [45]. The *FASN* mRNA expression in our study was not affected in the fat pad (Table 6), where it has been shown to be highly expressed [54]. *FASN* expression has been positively linked with cell proliferation and rapid development, which is the case of the 42-day-old broiler chickens examined here. Thus, a plausible explanation for the increased *FASN* expression observed could be that feed alone cannot meet the FA demands of actively proliferating tissues such as muscles, and as a consequence *FASN* expression rises [55]. 

Adiponectin is an adipokine produced mainly in the abdominal adipose tissue (fat pad), but also in the skeletal muscle [56]. It is involved in many biological processes and plays a protective role in diabetes, obesity, atherosclerosis and other metabolic deregulations. Increased levels of plasma adiponectin and mRNA expression in the fat pad have been detected in response to hesperidin and naringin supplementation [57,58,59]. Consistently with these findings, and in line with hesperidin’s beneficial effect on metabolic disorders and its anti-inflammatory properties, we detected increased *ADIPOQ* expression in the breast muscle in the E1 treatment group. 

Meat’s oxidative stability has been shown to improve as a result of hesperidin or naringin dietary supplementation [11]. This effect could be attributed to the improvement of antioxidant defense status of the tissues. Indeed, we observed a significant upregulation of glutathione reductase (*GSR*) gene expression in response to increasing naringin supplementation (Figure 1 and Supplementary Table S2). Hepatic *GSR* expression was also increased in hesperidin-supplemented animals, but the difference from the control group was not significant. This is in line with the oxidative stability data, which showed that the antioxidant activity of naringin was more pronounced compared to hesperidin [11]. 

GSR is an enzyme that plays an important role in resisting oxidative stress [60]. It catalyzes the reduction of oxidized glutathione (GSSG) to the reduced glutathione (GSH), which is an essential molecule in resisting oxidative stress and preserving the reducing environment of all tissues. GSH can act as a scavenger for hydroxyl radicals, singlet oxygen, and various electrophiles [61]. The GSSG/GSH ratio present in the cell is important for maintaining the oxidative balance of the cell [62]. It is crucial that the cell reserves high levels of GSH and a low level of GSSG, and this ratio is regulated by GSR. Furthermore, GSH plays a pivotal role in the clearance and metabolism of xenobiotics, acts as a cofactor in specific detoxifying enzymes, participates in transport, and restores antioxidants such as vitamins C and E to their active forms [63]. It can either scavenge hydroxyl radicals and singlet oxygen non enzymatically, or serve as an electron donor to several enzymes involved in reactive oxygen species (ROS) detoxification. Increasing GSH tissue levels may also provide benefits in terms of the improved meat quality of livestock animals. Post-mortem lipid hydroperoxidation and protein oxidation (e.g., oxymyoglobin to metmyoglobin) have significant effects on meat tenderness and color [64,65,66]. GSH as an antioxidant may play a considerable role in preserving the shelf life and quality of meat products [67]. This role may be compared with that of vitamin E [68].

Consistent with our finding, Kapoor et al. [69] showed that the in vitro addition of naringenin to glucose-stressed rat hepatocytes could prevent the generation of ROS and the decline in the cells’ antioxidant defense. In particular, they showed that naringenin restored GR expression in glucose-stressed rat hepatocytes to control levels. 

Although vitamin E supplementation also improved oxidative stability, as expected [11], no effect was observed on the hepatic expression of any of the antioxidant genes assessed here. This suggests that there are differences in the molecular mechanisms underlying the antioxidant activity of vitamin E and the two citrus flavanones. It could be argued that VE acts mainly via the direct scavenging of free radicals, while hesperidin and naringin also exert their antioxidant function via gene regulation, or alternatively that vitamin E regulates the expressions of genes not included in the array used in the present study. 

## 5. Conclusions

In conclusion, hesperidin and naringin had beneficial effects on broiler meat’s health-promoting properties. They improved fatty acid profile, by reducing SFA and increasing PUFA content, and beneficially affected the AI and TI indices in breast muscle and the abdominal fat pad. Low levels of supplementation (0.75 g per kg feed) were sufficient for both compounds to exert their beneficial effects. The effects of naringin on FA are likely to be mediated by an increase in FA β-oxidation via the modulation of the expression of *PPARα* and *ACOX1* in the liver. In the case of hesperidin, a role for *FASN* and *ADIPOQ* gene expression modulation in the breast muscle is indicated. Furthermore, the antioxidant properties of citrus flavanones observed in broilers could be attributed, at least partly, to the regulation of antioxidant defense genes, such as *GSR*, which was found here to be significantly affected by naringin supplementation. 

## Figures and Tables

**Table 1:** Genes included in the quantitative (q)PCR array.

Gene/Assay Symbol	Gene/Assay Description	Unigene	GeneBank	Gene Function
*CAT*	Catalase	Gga.1183	NM_001031215	Antioxidant
*DIO1*	Deiodinase, Iodothyronine deiodinase Type I	Gga.553	NM_001097614	
*DIO2*	Deiodinase, Iodothyronine deiodinase Type II	Gga.51485	NM_204114	
*GPX1*	Glutathione peroxidase 1	Gga.1465	NM_001277853	
*GPX4*	Glutathione peroxidase 4	Gga.107	XM_003642871	
*GSR*	Glutathione Reductase	Gga.34900	XM_001235016	
*SOD1*	CuZn Superoxide Dismutase	Gga.3346	NM_205064	
*SOD2*	Mn Superoxide Dismutase	Gga.937	NM_204211	
*SOD3*	extracellular Cu-Zn-Superoxide Dismutase	Gga.1128	XM_420760	
*TXNRD1*	Thioredoxin reductase Type I	Gga.4380	NM_001030762	
*TXNRD2*	Thioredoxin reductase Type II	Gga.29425	NM_001122691	
*BCL2*	B-cell CLL/lymphoma 2	Gga.42172	NM_205339	Apoptosis
*CASP3*	Caspase 3, apoptosis-related cysteine peptidase	Gga.4346	NM_204725	
*CASP8*	Caspase 8, apoptosis-related cysteine peptidase	Gga.2451	NM_204592	
*CASP9*	Caspase 9, apoptosis-related cysteine peptidase	Gga.4116	XM_424580	
*TMBIM1*	Transmembrane BAX inhibitor motif containing 1	Gga.7211	XM_422067	
*ACOX1*	Acyl-CoA oxidase 1, palmitoyl	Gga.39153	NM_001006205	Fatty Acid Metabolism
*CPT1A*	Carnitine palmitoyltransferase 1A (liver)	Gga.9299	NM_001012898	
*FASN*	Fatty acid synthase	Gga.8951	NM_205155	
*PPARA*	Peroxisome proliferator-activated receptor alpha	Gga.4006	NM_001001464	
*PPARG*	Peroxisome proliferator-activated receptor gamma	Gga.3858	NM_001001460	
*PPARGC1A*	Peroxisome proliferator-activated receptor gamma, coactivator 1 alpha	Gga.22894	NM_001006457	
*SCD*	Stearoyl-CoA desaturase	Gga.17055	NM_204890	
*SREBF1*	Sterol regulatory element binding transcription factor 1	Gga.51495	NM_204126	
*LDLR*	Low density lipoprotein receptor	Gga.8517	NM_204452	Lipid metabolism
*LPL*	Lipoprotein lipase	Gga.1152	NM_205282	
*ACACA*	acetyl-CoA carboxylase alpha	Gga.1480	NM_205505	Metabolism
*GCK*	Similar to glucokinase	Gga.48051	XM_004949993	
*IL10*	Interleukin 10	Gga.46641	NM_001004414	pro-inflammatory
*IL1B*	Interleukin 1, beta	Gga.19	NM_204524	
*IL2*	Interleukin 2	Gga.4946	NM_204153	
*IL6*	Interleukin 6 (interferon, beta 2)	Gga.2769	NM_204628	
*LITAF*	lipopolysaccharide-induced tumor necrosis factor-alpha factor homolog	Gga.3383	NM_204267	
*NOS2*	Inducible Nitric Oxide synthase	Gga.3327	NM_204961	
*PTGS2*	Prostaglandin-endoperoxide synthase 2	Gga.4401	NM_001167718	
*SMAD3*	SMAD family member 3	Gga.28197	NM_204475	
*ACTB*	Actin, beta	Gga.43416	NM_205518	Housekeeping
*H6PD*	Hexose-6-phosphate dehydrogenase	Gga.50291	XM_425746	
*HMBS*	Hydroxymethylbilane synthase	Gga.8480	XM_417846	
*RPL4*	Ribosomal protein L4	Gga.4523	NM_001007479	
*UBC*	Ubiquitin C	Gga.39142	XM_001234599	
*GGDC*	genomic DNA contamination negative control			
*RTC*	reverse transcription positive control			
*PPC*	PCR-positive control			

**Table 2:** Effect of hesperidin (E), naringin (N) and vitamin E (VE) on intramuscular fat content in *pectoralis major* (breast) and *biceps femoris* (thigh).

	Treatment							*p*-Value	*p*-Linear	
% Intramuscular Fat	C	E1	E2	N1	N2	VE	SEM		C-E1-E2	C-N1-N2
Breast	1.52	1.38	1.68	1.15	1.42	1.17	0.15	NS	NS	NS
Thigh	5.24	5.22	4.85	4.55	5.12	5.24	0.34	NS	NS	NS

C (no supplementation), E1 (0.75 g of hesperidin per kg of feed), E2 (1.5 g hesperidin/kg feed), N1 (0.75 g naringin/kg feed), N2 (1.5 g naringin/kg feed) and VE (0.2 g a-tocopheryl acetate/kg feed). Significance of treatment (*p*) and linear dose–response to E and N (*p*-linear) are shown. NS: not significant.

**Table 3:** Effect of hesperidin (E), naringin (N) and vitamin E (VE) on intramuscular fat content, fatty acid profile and atherogenicity (AI) and thrombogenicity (TI) indices in the pectoralis major breast muscle.

Tissue: Breast	Treatment (LSM)								*p*-Linear	
Parameter	C	E1	E2	N1	N2	VE	SEM	*p*-Value	C-E1-E2	C-N1-N2
FA (% of Total)										
C6:0	0.018	0.006 ^C^	0.014	0.007	0.011	0.012	0.0027	*	NS	NS
C10:0	0.016	0.017	0.018	0.012	0.011	0.017	0.0032	NS	NS	NS
C12:0	0.018	0.015	0.021	0.014	0.017	0.020	0.0030	NS	NS	NS
C14:0	0.45	0.44	0.44	0.42	0.43	0.42	0.01	NS	NS	NS
C14:1	0.071	0.072	0.064	0.061	0.070	0.060	0.0044	NS	NS	NS
C15:0	0.080	0.076	0.082	0.082	0.080	0.079	0.0028	NS	NS	NS
C16:0	25.60	24.33 ^C^	24.74	24.81	24.29 ^C^	24.24 ^C^	0.28	**	*	**
C16:1	3.22	3.29	3.01	2.91	3.05	2.93	0.16	NS	NS	NS
C17:0	0.112	0.102	0.113	0.114	0.110	0.114	0.005	NS	NS	NS
C17:1	0.071	0.070	0.070	0.068	0.069	0.065	0.004	NS	NS	NS
C18:0	8.14	6.98 ^C,VE^	6.88 ^C,VE^	7.14 ^C,VE^	7.48	7.87	0.17	****	****	*
C18:1	33.68	33.45	33.12	33.28	33.53	33.88	0.36	NS	NS	NS
C18:2n-6	22.89	25.06 ^C^	25.69 ^C,VE^	25.39 ^C,VE^	24.65 ^C^	23.66	0.38	****	****	**
C18:3n-3	1.71	1.81 ^VE^	1.79	1.74	1.76	1.65	0.04	*	0.1	NS
C20:3n-6	0.435	0.352	0.417	0.437	0.410	0.395	0.038	NS	NS	NS
C20:4n-6	0.202	0.230	0.203	0.215	0.193	0.193	0.009	*	NS	NS
C20:5n-3	2.03	2.15 ^VE^	2.17 ^VE^	2.17 ^VE^	2.53	3.08 ^C^	0.20	**	NS	0.07
C22:5n-3	0.028	0.251 ^C,VE^	0.022	0.006	0.003	0.054	0.019	****	NS	*
C22:6 n-3	1.29	1.42	1.17	1.15	1.32	1.32	0.10	NS	NS	NS
SFA	34.42	31.95 ^C^	32.29 ^C^	32.59 ^C^	32.42 ^C^	32.76 ^C^	0.34	****	***	**
MUFA	37.04	36.88	36.26	36.32	36.72	36.94	0.45	NS	NS	NS
PUFA	28.57	31.26 ^C^	31.46 ^C^	31.11 ^C^	30.87 ^C^	30.35	0.48	***	***	**
PUFA/SFA	0.831	0.980 ^C^	0.977 ^C^	0.956 ^C^	0.953 ^C^	0.927 ^C^	0.021	****	****	**
n-6	23.09	25.29 ^C^	25.89 ^C,VE^	25.61 ^C,VE^	24.84 ^C^	23.85	0.37	****	****	**
n-3	5.05	5.62	5.15 ^VE^	5.06 ^VE^	5.62	6.10 ^C^	0.21	**	NS	NS
n-6/n-3	4.62	4.54	5.10 ^VE^	5.14 ^VE^	4.44	3.98	0.18	****	NS	NS
AI	0.418	0.383 ^C^	0.392	0.393 ^C^	0.385 ^C^	0.386 ^C^	0.006	**	**	**
TI	0.753	0.660 ^C^	0.689 ^C^	0.701	0.674 ^C^	0.667 ^C^	0.015	***	**	**

C (no supplementation), E1 (0.75 g of hesperidin per kg of feed), E2 (1.5 g hesperidin/kg feed), N1 (0.75 g naringin/kg feed), N2 (1.5 g naringin/kg feed) and VE (0.2 g a-tocopheryl acetate/kg feed). Significance of treatment (*p*) and linear dose-response to E and N (*p*-linear) are shown. ^C^: Means differ significantly from C (*p* < 0.05). ^VE^: Means differ significantly from VE (*p* < 0.05). * *p* < 0.05, ** *p* < 0.01, *** *p* < 0.001, **** *p* < 0.0001. NS: not significant.

**Table 4:** Effect of hesperidin (E), naringin (N) and vitamin E (VE) on intramuscular fat content, fatty acid profile and atherogenicity (AI) and thrombogenicity (TI) indices in the *biceps femoris* thigh muscle.

Tissue: Thigh	Treatment								*p*-Linear	
Parameter	C	E1	E2	N1	N2	VE	SEM	*p*-Value	C-E1-E2	C-N1-N2
FA (% of Total)										
C6:0	0.004	0.010 ^C^	0.005	0.004	0.007	0.006	0.001	*	NS	NS
C10:0	0.008	0.008	0.007	0.008	0.008	0.007	0.001	NS	NS	NS
C12:0	0.022	0.022	0.021	0.022	0.022	0.020	0.001	NS	NS	NS
C14:0	0.468	0.481	0.467	0.466	0.486 ^VE^	0.442	0.010	*	NS	NS
C14:1	0.088	0.091	0.080	0.079	0.091	0.077	0.005	NS	NS	NS
C15:0	0.075	0.079	0.080	0.082	0.080	0.078	0.003	NS	NS	NS
C16:0	23.97	23.94	24.24	24.06	23.89	23.27	0.28	NS	NS	NS
C16:1	4.250	4.403	3.943	3.956	4.509	3.956	0.198	NS	NS	NS
C17:0	0.080	0.083	0.090	0.089	0.083	0.087	0.006	NS	NS	NS
C17:1	0.107	0.106	0.102	0.110	0.112	0.106	0.005	NS	NS	NS
C18:0	5.438	5.413	5.599	5.038	5.324	5.649	0.225	NS	NS	NS
C18:1	35.89	36.14	35.46	35.15	35.91	37.34	0.54	^	NS	NS
C18:2n-6	25.34	25.41	25.77	26.85	25.64	25.16	0.54	NS	NS	NS
C18:3n-3	2.094	2.083	2.135	2.151	2.118	2.064	0.035	NS	NS	NS
C20:3n-6	0.139	0.148	0.159	0.163	0.155	0.143	0.010	NS	NS	NS
C20:4n-6	0.228	0.225	0.222	0.224	0.225	0.221	0.008	NS	NS	NS
C20:5n-3	0.963	0.956	1.057	1.146	1.007	1.046	0.081	NS	NS	NS
C22:5n-3	0.046	0.057	0.046	0.004	0.009	0.020	0.010	**	NS	**
C22:6n-3	0.821	0.374 ^C^	0.567 ^C,VE^	0.403 ^C^	0.331 ^C^	0.315 ^C^	0.041	****	**	****
SFA	30.06	30.03	30.51	29.76	29.89	29.55	0.32	NS	NS	NS
MUFA	40.33	40.74	39.59	39.29	40.62	41.48	0.63	NS	NS	NS
PUFA	29.63	29.25	29.96	30.94	29.48	28.97	0.64	NS	NS	NS
PUFA/SFA	0.988	0.975	0.984	1.040	0.988	0.983	0.027	NS	NS	NS
n-6	25.56	25.63	25.99	27.07	25.86	25.38	0.54	NS	NS	NS
n-3	3.923	3.470	3.805	3.704	3.464	3.446	0.118	*	NS	^
n-6/n-3	6.578	7.412 ^C^	6.859	7.329 ^C^	7.500 ^C^	7.400 ^C^	0.156	***	NS	**
AI	0.370	0.370	0.376	0.370	0.369	0.356	0.006	NS	NS	NS
TI	0.667	0.683	0.684	0.666	0.679	0.670	0.012	NS	NS	NS

C (no supplementation), E1 (0.75 g of hesperidin per kg of feed), E2 (1.5 g hesperidin/kg feed), N1 (0.75 g naringin/kg feed), N2 (1.5 g naringin/kg feed) and VE (0.2 g a-tocopheryl acetate/kg feed). Significance of treatment (P) and linear dose–response to E and N (*p*-linear) are shown. ^C^: Means differ significantly from C (*p* < 0.05). ^VE^: Means differ significantly from VE (*p* < 0.05). * *p* < 0.05, ** *p* < 0.01, *** *p* < 0.001, **** *p* < 0.0001, ^ 0.10 > *p* > 0.05. NS: not significant.

**Table 5:** Effect of hesperidin (E), naringin (N) and vitamin E (VE) on fatty acid (FA) profile and atherogenicity (AI) and thrombogenicity (TI) indices in the abdominal adipose tissue.

Tissue: Fat Pad	Treatment (LSM)							*p*-Value	*p*-Linear	
Parameter	C	E1	E2	N1	N2	VE	SEM	Treatment	C-E1-E2	C-N1-N2
FA (% of Total)										
C10:0	0.006	0.008	0.007	0.008	0.008	0.008	0.001	NS	NS	NS
C12:0	0.021	0.023	0.022	0.027	0.022	0.021	0.002	NS	NS	NS
C14:0	0.471	0.481	0.475	0.488	0.479	0.447	0.016	NS	NS	NS
C14:1	0.096	0.096	0.087	0.082	0.096	0.082	0.007	NS	NS	NS
C15:0	0.076	0.074	0.074	0.073	0.065	0.071	0.005	NS	NS	NS
C16:0	24.16	23.22	23.03	22.94	23.15	23.41	0.35	NS	^	^
C16:1	4.756	4.548	4.109	4.406	4.536	4.083	0.269	NS	NS	NS
C17:0	0.069	0.072	0.079	0.074	0.071	0.075	0.007	NS	NS	NS
C17:1	0.108	0.115	0.108	0.109	0.119	0.122	0.006	NS	NS	NS
C18:0	4.348	4.406	4.500	4.492	4.290	4.213	0.269	NS	NS	NS
C18:1	38.88	37.16 ^C^	37.57 ^C^	37.56 ^C^	37.81	36.90 ^C^	0.31	***	*	^
C18:2n-6	23.73	25.64 ^C^	26.06 ^C^	26.17 ^C^	25.93 ^C^	26.94 ^C^	0.42	****	**	**
C18:3n-3	2.164	2.227	2.518 ^C^	2.307	2.306	2.323	0.062	**	***	*
C20:3n-6	0.078	0.104	0.190	0.086	0.112	0.097	0.029	NS	**	NS
C20:4n-6	0.230	0.467 ^C,VE^	0.284	0.236	0.213	0.242	0.046	**	NS	NS
C20:5n-3	0.299	0.615	0.323	0.498	0.278	0.447	0.107	NS	NS	NS
C22:5n-3	0.235	0.242	0.332	0.224	0.283	0.262	0.046	NS	^	NS
C22:6n-3	0.279	0.480	0.262	0.238	0.220	0.247	0.079	NS	NS	NS
SFA	29.15	28.29	28.19	28.10	28.09	28.24	0.38	NS	NS	^
MUFA	43.84	41.92 ^C^	41.87 ^C^	42.15	42.56	41.19 ^C^	0.46	**	**	NS
PUFA	27.01	29.77 ^C^	29.97 ^C^	29.76 ^C^	29.34 ^C^	30.56 ^C^	0.46	****	***	**
PUFA/SFA	0.931	1.055 ^C^	1.065 ^C^	1.061 ^C^	1.048 ^C^	1.084 ^C^	0.025	**	**	**
n-6	23.97	26.10 ^C^	26.34 ^C^	26.40 ^C^	26.14 ^C^	27.18 ^C^	0.43	****	**	**
n-3	2.978	3.564	3.434	3.267	3.088	3.279	0.164	NS	^	NS
n-6/n-3	8.245	7.718	7.731	8.136	8.586	8.399	0.382	NS	NS	NS
AI	0.368	0.351	0.347	0.347	0.349	0.352	0.007	NS	^	^
TI	0.676	0.629	0.630	0.632	0.640	0.637	0.014	NS	*	NS

Significance of treatment (P) and linear dose–response to E and N (*p*-linear) are shown. C (no supplementation), E1 (0.75 g of hesperidin per kg of feed), E2 (1.5 g hesperidin/kg feed), N1 (0.75 g naringin/kg feed), N2 (1.5 g naringin/kg feed) and VE (0.2 g a-tocopheryl acetate/kg feed). ^C^: Means differ significantly from C (*p* < 0.05). ^VE^: Means differ significantly from VE (*p* < 0.05). * *p* < 0.05, ** *p* < 0.01, *** *p* < 0.001, **** *p* < 0.0001, ^ 0.10 > *p* > 0.05. NS: not significant.

**Table 6:** Effects of hesperidin (E), naringin (N) and vitamin E (VE) on mean expression of adiponectin (*ADIPOQ*), fatty acid synthase (*FASN*) and peroxisome proliferator-activated receptor gamma (*PPARγ*) in the breast *pectoralis major* (breast) muscle and abdominal adipose tissue (fat pad).

	Treatment Mean ± SEM						*p*-Value			*p*-Linear	
	C	E1	E2	N1	N2	VE	E	N	VE	C-E1-E2	C-N1-N2
Breast muscle											
*ADIPOQ*	1 ± 0.11	1.28 ± 0.06	1.11 ± 0.08	1.13 ± 0.08	0.87 ± 0.04	1.00 ± 0.09	*	^	NS	NS	NS
*FASN*	1 ± 0.09	1.26 ± 0.12	1.77 ± 0.19	1.36 ± 0.08	1.37 ± 0.12	1.09 ± 0.07	***	^	NS	****	**
*PPARγ*	1 ± 0.12	0.98 ± 0.09	1.09 ± 0.17	1.05 ± 0.07	0.93 ± 0.07	0.92 ± 0.09	NS	NS	NS	NS	NS
Fat pad											
*ADIPOQ*	1 ± 0.08	0.96 ± 0.25	1.46 ± 0.29	1.11 ± 0.32	1.06 ± 0.3	1.09 ± 0.18	NS	NS	NS	NS	NS
*FASN*	1 ± 0.06	1.82 ± 0.56	1.15 ± 0.19	1.29 ± 0.27	1.09 ± 0.22	1.33 ± 0.18	^	NS	NS	NS	NS
*PPARγ*	1 ± 0.17	1.27 ± 0.47	1.33 ± 0.17	0.8 ± 0.22	0.74 ± 0.17	1.01 ± 0.28	NS	NS	NS	NS	NS

Expression is relative to the control group. Significance of hesperidin (E), naringin (N) and vitamin E (VE) treatment effects (*p*-value) and linear dose–response relationship of E and N (*p*-linear) are shown. C (no supplementation), E1 (0.75 g of hesperidin per kg of feed), E2 (1.5 g hesperidin/kg feed), N1 (0.75 g naringin/kg feed), N2 (1.5 g naringin/kg feed) and VE (0.2 g a-tocopheryl acetate/kg feed). * *p* < 0.05, ** *p* < 0.01, *** *p* < 0.001, **** *p* < 0.0001, ^ 0.10 > *p* > 0.05. NS: not significant.

## Supplementary Materials

The following are available online at https://www.mdpi.com/article/10.3390/foods10040739/s1, Table S1: Review of genes found to be differentially regulated in the liver in response to hesperidin and/or naringin or their aglycones, hesperetin and naringenin, or to citrus fruit extracts containing them. Table S2: Effects of hesperidin (E), naringin (N) and vitamin E (VE) on mean expression of selected genes included in the qPCR array assay, involved in lipid metabolism, antioxidant defense and anti-inflammatory immune regulation.
